# Methylation associated with long- or short-term survival in glioblastoma patients from the Nordic phase 3 trial

**DOI:** 10.3389/fgene.2022.934519

**Published:** 2022-08-25

**Authors:** Małgorzata Łysiak, Jyotirmoy Das, Annika Malmström, Peter Söderkvist

**Affiliations:** ^1^ Department of Biomedical and Clinical Sciences, Linköping University, Linköping, Sweden; ^2^ Bioinformatics Unit (Core Facility), Department of Biomedical and Clinical Sciences, Linköping University, Linköping, Sweden; ^3^ Clinical Genomics Linköping, SciLife Laboratory, Department of Biomedical and Clinical Sciences, Linköping University, Linköping, Sweden; ^4^ Department of Advanced Home Care, Linköping University, Linköping, Sweden

**Keywords:** glioblastoma, methylation, long-term survival, short-term survival, temozolomide, radiotherapy, epigenetic age

## Abstract

Patients with glioblastoma (GBM) have a poor outcome, but even among patients receiving the same therapies and with good prognostic factors, one can find those with exceptionally short and long survival. From the Nordic trial, which randomized GBM patients of 60 years or older between two radiotherapy arms (60 Gy or 34 Gy) or temozolomide (TMZ), we selected 59 with good prognostic factors. These selected GBM patients were equally distributed according to treatment and MGMT promoter methylation status but had long or short survival. Methylation profiling with the Illumina Infinium Methylation EPIC BeadChip arrays was performed and utilized for methylation-based CNS tumor classification, and pathway enrichment analysis of differentially methylated CpG sites (DMCs), as well as calculation of epigenetic age acceleration with three different algorithms, to compare the long and short survival groups. Samples identified by the classifier as non-GBM *IDH* wildtype were excluded. DMCs between long- and short-term survivors were found in patients with methylated *MGMT* promoter treated with TMZ (123,510), those with unmethylated *MGMT* treated with 60Gy radiotherapy (4,086), and with methylated *MGMT* promoter treated with 34Gy radiotherapy (39,649). Long-term survivors with methylated *MGMT* promoter treated with TMZ exhibited hypermethylation of the Wnt signaling and the platelet activation, signaling, and aggregation pathways. The joint analysis of radiotherapy arms revealed 319 DMCs between long- and short-term survivors with unmethylated *MGMT* and none for samples with methylated *MGMT* promoter. An analysis comparing epigenetic age acceleration between patients with long- and short-term survival across all treatment arms showed a decreased epigenetic age acceleration for the latter. We identified DMCs for both TMZ and RT-treated patients and epigenetic age acceleration as a potential prognostic marker, but further systematic analysis of larger patient cohorts is necessary for confirmation of their prognostic and/or predictive properties.

## Introduction

Glioblastoma (GBM) remains the most common and deadliest among gliomas. The peak incidence is in individuals above 65 years old, and only about 5.6% of patients reach five-year survival (Ostrom et al., 2018). Unfortunately, the treatment options are also limited, and so far, efforts have only provided modest survival benefits, leaving the median survival at 1.2 years (Molinaro et al., 2019; Weller et al., 2020). Standard therapy for GBM patients consists of gross total resection, if feasible, followed by concomitant radiotherapy (RT) and chemotherapy with the alkylating agent temozolomide (TMZ) and additional six cycles of TMZ (Weller et al., 2021). For fit patients, additional treatment with tumor-treating fields is an option, further prolonging survival for the selected group by nearly 5 months (Stupp et al., 2017). Choosing the best possible treatment is crucial, especially for elderly patients often burdened by comorbidities, where the combined treatment is not expected to be tolerated. The known positive prognostic factors include the extent of surgery, age, performance status, and sex of the patient (Weller et al., 2020). Thus far, the only predictive biomarker associated with response to TMZ treatment is the methylation status of the promoter of the O^6^-methylguanine-DNA-methyltransferase (*MGMT*) gene, and methylated *MGMT* (m-MGMT) is associated with better overall survival (OS) (Stupp et al., 2007; Malmstrom et al., 2012), although varying responses among patients are still observed.

Notably, the importance of epigenetic changes in gliomas has been emphasized by recent discoveries (Etcheverry et al., 2010; Capper et al., 2018; Chai et al., 2019; Wu et al., 2020). The current classification of brain tumors incorporates molecular biomarkers, namely., mutations of isocitrate dehydrogenase 1 or 2 (*IDH1/2*) and 1p/19q codeletion (Louis et al., 2016). Methylome profiling presents a promising alternative, with further refinement and subclassification (Capper et al., 2018). Another example is the glioma cytosine–phosphate–guanine (CpG) island methylator phenotype (G-CIMP), which entails genome-wide hypermethylation of the CpG islands. The G-CIMP profile is tightly associated with *IDH1/2* mutations and better outcomes for patients with *IDH*-mutated gliomas (Noushmehr et al., 2010; Malta et al., 2018; Tesileanu et al., 2021). There are also other indications that methylome profiling could aid in selection of patients with better prognosis within the same diagnostic entity, for e.g., by using the methylation differences found in short- and long-term survivors (STS and LTS, respectively) with GBM (Shinawi et al., 2013; Ma et al., 2015; Klughammer et al., 2018). Moreover, changes in the methylation profiles between primary and recurrent GBM have been reported, with common occurrence of switches between methylation subclasses found at progression (Klughammer et al., 2018).

Age is one of the prognostic factors, and younger GBM patients are characterized by better outcomes (Ostrom et al., 2018; Weller et al., 2020). Aging is also reflected in the methylation state of the genome, the so-called epigenetic age (Lu et al., 2019). Methylation of specific CpG sites undergoes age-dependent changes, which can be quantified and expressed through the epigenetic age (Horvath, 2013). Acceleration of the epigenetic age, which is the difference between the epigenetic age and chronological age, has been reported in many diseases, such as Alzheimer’s (Levine et al., 2015) or Parkinson’s disease (Horvath and Ritz, 2015). It has also shown to be associated with cancer, mortality in cardiovascular diseases (Lin and Wagner, 2015; Perna et al., 2016), and patients outcome in gliomas (Liao et al., 2018; Zheng et al., 2020).

In the Nordic trial, patients of 60 years or older diagnosed with GBM were randomized between two different fractionation regimens of RT or TMZ treatment alone (Malmstrom et al., 2012). Constituting a unique cohort with the possibility to investigate the role of methylation profiles in relation to RT and TMZ separately, we decided to analyze the global methylation status of tumors from LTS and STS using Illumina EPIC bead arrays. The aim of this research was to identify potential methylation-based biomarkers or profiles related to treatment and outcome.

## Materials and methods

### Patients

**FIGURE 1:**
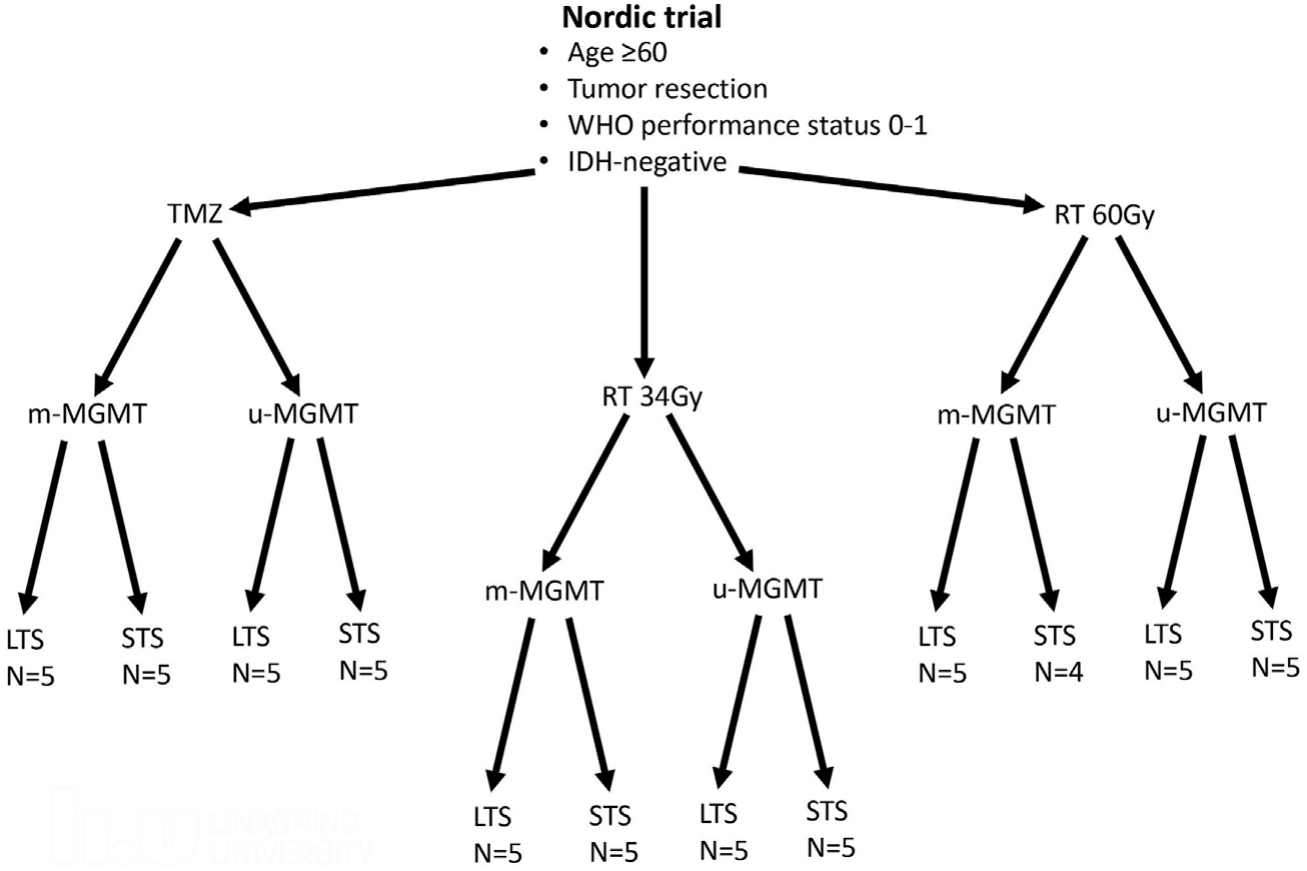
Patients from the Nordic trial included in the study. Patients with good prognostic factors were selected from each treatment arm (temozolomide -TMZ, radiotherapy 34Gy-RT 34Gy, and radiotherapy 60Gy- RT60Gy). Half of the tumors had methylated *MGMT* promoter (m-MGMT), and half had unmethylated *MGMT* promoter (u-MGMT). These patients were further divided into long-term survivors (LTS) and short-term survivors (STS). N, number of patients.

All patients included in this study were participants of the Nordic-randomized, phase 3 trial registered under number ISRCTN81470623 (Malmstrom et al., 2012), which compared three treatment modalities for newly diagnosed GBM patients with age 60 and above (TMZ vs. standard RT 60Gy vs. hypofractionated RT 34Gy). First, from the entire trial, we selected patients with available formalin-fixed paraffin-embedded (FFPE) tumor tissue. From each of the three treatment arms (TMZ, 34, and 60Gy), we chose patients with immunohistochemically confirmed IDH1 wildtype status and characterized the patients by two good prognostic factors: tumor resection and WHO performance status 0–1 (Malmstrom et al., 2012). Next, samples within each treatment arm were stratified based on their *MGMT* promoter methylation status (methylated *MGMT* promoter, m-MGMT; unmethylated *MGMT* promoter, u-MGMT) and ranked based on the survival. From each group, we selected five patients with the longest and five with the shortest survival, so that the treatment modality, MGMT status, and survival group were represented by an equal number of patients (Figure 1 and Table 1). The *MGMT* methylation status was analyzed with the MDxHealth method, Liège, Belgium, as mentioned in Malmstrom et al. (2012). Ethical approval for the Nordic trial and molecular analyses were previously obtained (99086, M11-06 T40-09, and 2011/32–32).

**TABLE 1:** Patient characteristics.

Treatment	Survival group	MGMT status	*N*	Male	Mean age at diagnosis (range) [years]	Median survival (range) [months]
TMZ	LTS	Methylated	5	3	64 (60–69)	20.1 (13.7–125.9)
		Unmethylated	5	3	72 (67–77)	13.3 (9.9–18.9)
	STS	Methylated	5	2	70.6 (67–78)	9.2 (3.5–10.4)
		Unmethylated	5	3	66.8 (64–69)	2.9 (2.1–3.8)
34Gy	LTS	Methylated	5	1	69.2 (63–77)	14.6 (12.6–24.3)
		Unmethylated	5	3	72.4 (69–81)	14.6 (12.6–35.7)
	STS	Methylated	5	2	72.4 (65–77)	4 (1.3–5.5)
		Unmethylated	5	2	68.6 (64–74)	4.6 (3.3–5.1)
60Gy	LTS	Methylated	5	2	64.2 (60–72)	14 (12.1–16.6)
		Unmethylated	5	4	64.6 (60–70)	17.9 (15.6–33.4)
	STS	Methylated	4	3	69.3 (62–73)	7.8 (6.6–10.1)
		Unmethylated	5	4	69.2 (65–74)	1.7 (1.1–4.2)

### DNA methylation analysis

DNA was extracted from two tumor tissue sections of 10 µm each, using the Maxwell FFPE DNA Purification Kit (Promega) according to the manufacturer’s protocol but with a double amount of proteinase K (40 mg/ml). DNA quantity and quality were checked using the NanoDrop ND-1000 spectrophotometer (ThermoFisher) and Quantus Fluorometer (Promega), as well as with the Infinium FFPE QC Kit (Illumina). A total of 250–500 ng of DNA was subjected to bisulfite conversion using the EZ DNA methylation kit (Zymo Research), and genome-wide DNA methylation was assessed with the Infinium Methylation EPIC BeadChip Kit (Illumina) complemented by the Infinium HD FFPE DNA Restore Kit as per manufacturer’s protocol. The BeadChip arrays were scanned on the NextSeq 550 (Illumina), and DNA methylation data in the form of IDAT files (intensity data files that contain green and red signals from methylated and unmethylated CpG sites) were uploaded to the online classifier v11b4 (Capper et al., 2018), where *MGMT* methylation status was also assessed with the MGMT-STP27 algorithm (Bady et al., 2016).

### Differential methylation analysis

The IDAT files from Illumina Human Methylation EPIC arrays were also analyzed using R (v4.0.3) (R Core Team, 2021) and Bioconductor packages (v3.14) (Gentleman et al., 2004), for example., the Chip Analysis Methylation Pipeline (ChAMP) analysis package (v2.19.3) (Tian et al., 2017). The files were pre-processed in ChAMP to filter out CpGs with detection *p*-value >0.01, as well as SNP CpGs, unbound and multi-hit CpGs, and all CpGs from sex chromosomes. After filtration, a quality check was performed, and the files were normalized with the beta-mixture quantile normalization (BMIQ) function. The β- and M-values of the samples were calculated against each CpG per sample. Batch effects were corrected with the *runCombat* function. The differential methylation analysis was calculated with the linear modeling (*limFit*) and *eBayes* algorithm, comparing two groups from the phenotypic dataset, and singular value decomposition (SVD) analysis was performed to check for confounders (e.g., age and sex) (Supplementary Data, Supplementary Figure S2). The differential methylation analysis was performed first for all samples that were classified as GBM *IDH* wildtype. Then, after removal of patients that died due to causes other than tumor, such as infection, the same analysis was conducted for three samples from each group, i.e., three with the longest survival in LTS and three with the shortest survival in STS. The latter analysis found differentially methylated CpGs (DMCs) in the comparison of LTS vs. STS within each treatment arm with methylated or unmethylated *MGMT* promoter; hence, further analysis was based on these. The DMCs were considered significant at the Bonferroni–Hochberg corrected *p*-value (*p*-value_BH_) <0.05. The hierarchical cluster analysis was performed using the Euclidean distance within the *ape* package (v5.0) (Paradis and Schliep, 2019) in R.

### Structural annotation

We used *AnnotationDbi* package (v1.54.1) (Pagès et al., 2021) to annotate DMCs and in-house scripts to visualize their genomic distribution. The statistically significant DMCs (*p*-value_BH_ < 0.05) were used to create the volcano plot with the mean methylation difference (∆mmd) ≥|0.3|) using the *EnhancedVolcano* package (v1.10.0) (Blighe et al., 2021) in R. The cut-off score of ∆mmd was calculated using the β-value distribution of all samples with the mean ± 2SD (Supplementary Data, Supplementary Figure S3, and Supplementary Data, Supplementary Table S1). The R package *ComplexHeatmap* (v2.8.0) (Gu et al., 2016) was used to create the heatmap from individual β-values of DMCs (*p*-value_BH_ < 0.05; (∆mmd) ≥|0.3|).

### Pathway enrichment and correlation analysis

To reduce the number of DMCs, we first filtered out DMCs based on the genomic location, leaving only those from the 5′-untranslated region (5′-UTR) and transcription start site (TSS) regions (TSS200 and TSS1500). Furthermore, we applied the ∆mmd cut-off score (as described previously). The DMCs were converted to their respective official gene symbols (hereafter called DMGs, differentially methylated genes), and the list (without/with ∆mmd values) was used for the pathway enrichment analysis. The Reactome database (v78) (Griss et al., 2020) was applied to perform the gene set enrichment analysis using the c*lusterProfiler* package (v4.0.5) (Wu et al., 2021) in R (v4.1) with the default parameters setup (e.g., 1,000 permutations and *p*-value_BH_ < 0.05). The results were visualized using ggplot2 (v3.3.3) (Wickham, 2016) in-house script.

### Epigenetic age calculation

The epigenetic age was calculated for the same set of samples that were used in the differential methylation analysis (three samples from STS and LTS per group). We used methylation data obtained in this study and followed the methods published for three epigenetic clocks, namely, Horvath’s (Horvath, 2013), Hannum’s (Hannum et al., 2013), and PhenoAge (Levine et al., 2018). The epigenetic age acceleration was calculated as the difference between the epigenetic age and chronological age, given in years. Mean chronological and epigenetic ages were compared with two-tailed Student’s t-test, and a *p-*value <0.05 was considered significant. Calculations were performed with IBM SPSS (v.26).

## Results

### Methylation-based classification

Histological review in the primary analysis of the trial material classified 58 out of 59 samples included in this study as GBM grade 4, and one sample was classified as astrocytoma grade 3, but all samples were *IDH1* mutation-negative. Methylation data for all samples passed the quality control and were uploaded to the brain tumor classifier (https://www.molecularneuropathology.org/mnp) (Capper et al., 2018). Methylation-based classification placed most of the samples in the GBM *IDH* wildtype class, including the mentioned astrocytoma. One sample was classified as anaplastic pilocytic astrocytoma, and confirmative sequencing of *IDH1* and *IDH2* displayed the absence of mutation in accordance with the immunohistochemistry result. In three cases, samples were classified as “control tissue” probably due to low tumor cell content and were excluded. The analysis of methylation subclasses revealed that 21 samples belonged to only one subclass, with 2 being midline GBM. The remaining (n = 34) GBM *IDH* wildtype samples had two or three subclasses assigned to them. The *MGMT* methylation analysis results were concordant between the MDxHealth method and MGMT-STP27 for all but one sample, even for the samples that were classified as control tissue.

### Differential methylation analysis

We decided to only include samples, where progressive disease and/or death caused by the tumor had been reported because, especially in the STS groups, the true relationship between the tumor’s methylation profile and survival could be compromised. After removing cases where death was caused by co-morbidity or complications (e.g., infection and pulmonary embolism), we decided to include only three samples with extreme survival times from each treatment arm and MGMT group, as survival times, especially in the RT arms, which had relatively small spread. The survival differences between LTS and STS as Kaplan–Meier curves are shown in the Supplementary Data, Supplementary Figure S1.

**FIGURE 2:**
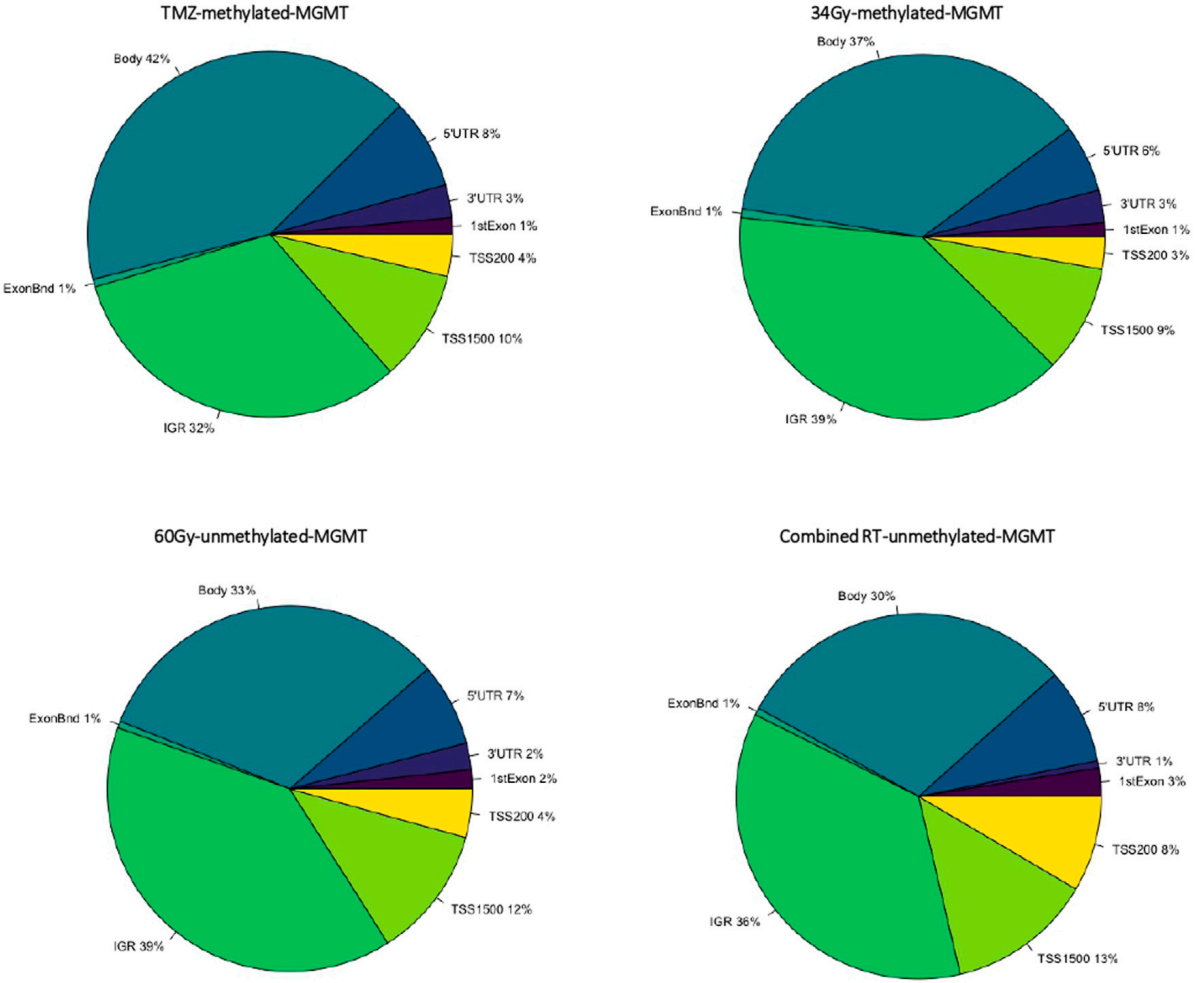
Pie charts representing the structural genomic distribution of DMCs discovered in samples with long (*n* = 3) and short (*n* = 3) survival within the different treatment arms and with specified *MGMT* promoter methylation status. TSS200, 200 bases upstream transcription start site; TSS1500, 1,500 bases upstream transcription start site; UTR, untranslated region; IGR, intergenic region; 60Gy34Gy, combined RT.

**FIGURE 3:**
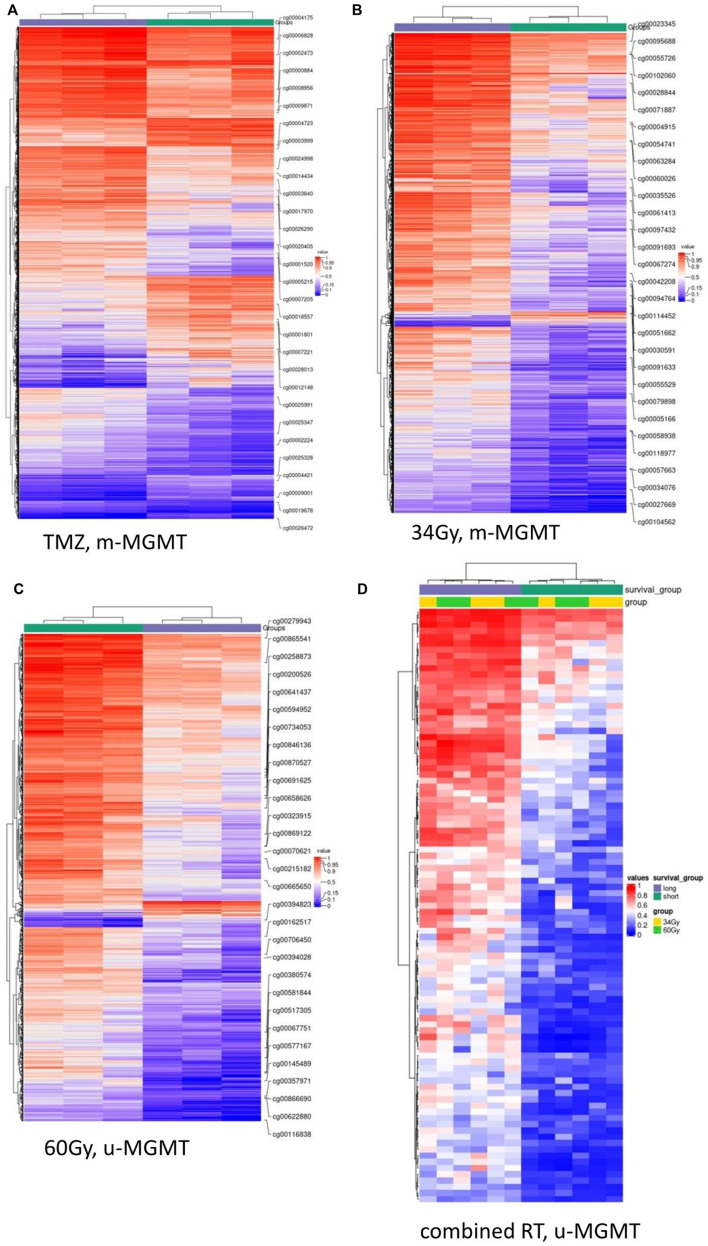
(Continued).

We compared LTS and STS samples in the separated treatment arms (TMZ, 34Gy, 60Gy, and combined RT), and within each treatment, we compared m-MGMT and u-MGMT samples separately and in combination. DMCs between LTS and STS were identified in the TMZ arm, m-MGMT; 34Gy, m-MGMT; 60Gy, u-MGMT, and in the combined RT arm, u-MGMT. The highest number of DMCs were found in the TMZ group (Supplementary Data, Supplementary Table S1). The cofactor analysis showed that the differential methylation analysis was not influenced by the included confounders (age, sex, and death by the tumor/progressive disease) (Supplementary Data, Supplementary Figure S2). Upon the structural annotation of DMCs, we found that they were similarly distributed throughout the genome (Figure 2), with the majority of DMCs found in the gene bodies and intergenic regions. Approximately 10% of DMCs were found in the TSS1500 and up to 8% in the 5′UTR, with both regions being of regulatory importance for gene expression due to the location of gene promoters. We reduced the number of DMCs for further analysis by filtering them out based on the genomic location (TSS1500, TSS200, and 5′-UTR) and ∆mmd cut-off values. Inspection of density plots created from filtered data revealed that the majority of DMCs were hypermethylated in LTS in the TMZ and 34Gy, m-MGMT groups and hypomethylated in LTS in the 60Gy, u-MGMT group. All DMCs were hypomethylated in the comparison of LTS vs. STS in the combined RT group with u-MGMT (Supplementary Data, Supplementary Figure S3). Next, we performed hierarchical cluster analysis on filtered DMCs (Figure 3). We observed that in the TMZ, m-MGMT group, data formed four clusters of DMCs between LTS and STS. We also found that for the joint RT group with u-MGMT, clusters separated LTS and STS but not the radiation doses (34 and 60Gy), emphasizing the importance of the treatment modality itself.

### Pathway enrichment analysis

**FIGURE 4:**
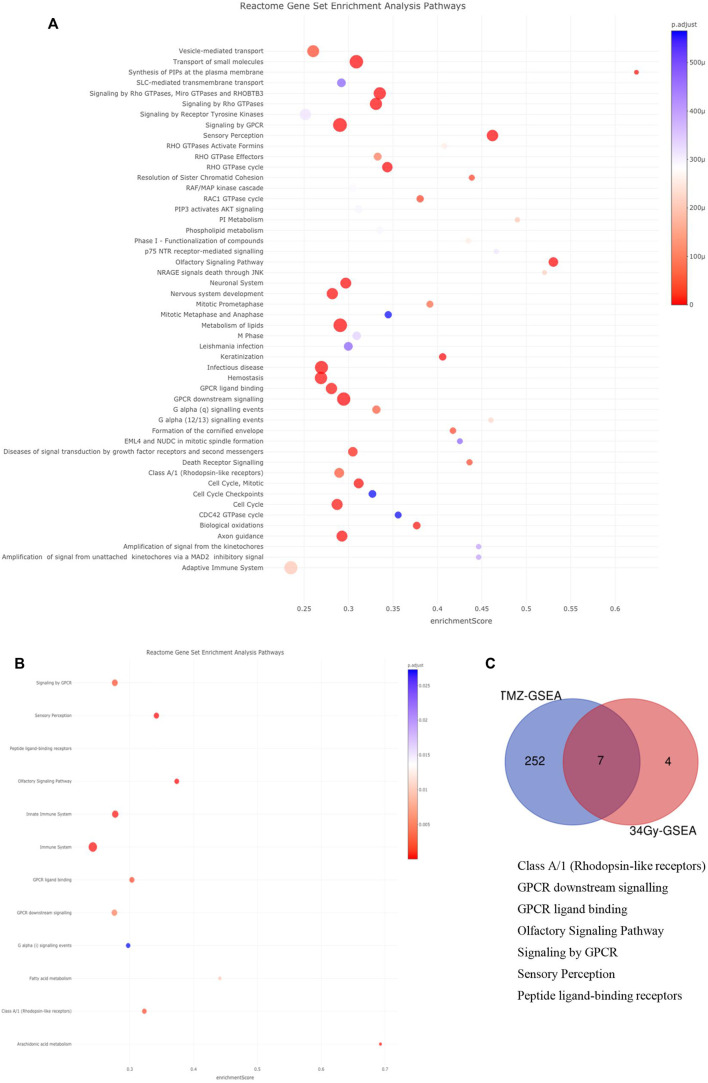
(Continued).

Next, we used the Reactome database to investigate pathway enrichment among the filtered DMGs, which were obtained from the annotated DMCs. There were four pathways enriched in the hypermethylated DMGs among LTS from the TMZ, m-MGMT group, namely, metabolism; platelet activation, signaling, and aggregation; signaling by WNT; and signal transduction. In the same group analysis (LTS from the TMZ, m-MGMT), we found 37 pathways enriched in the hypomethylated DMGs, for example., the immune system and Rho signaling pathways (Supplementary Data, Supplementary Table S2). In the hypermethylated DMGs in LTS in the treatment of 34Gy with m-MGMT, we found two enriched pathways (immune system and class B/2 secretin family receptors) (Supplementary Data, Supplementary Table S2). We did not find any enriched pathways for the remaining groups. Due to stringent filters applied initially to DMCs and a low number of detected enriched pathways, we decided to include DMCs removed by the ∆mmd cut-off values and repeat the analysis. Consequently, the number of enriched pathways increased, both for the TMZ, m-MGMT and 34Gy, m-MGMT groups (Figure 4), but no pathways were enriched in the 60Gy and combined RT groups. We compared the lists of enriched pathways and found seven that were common for the TMZ and 34Gy (m-MGMT) arms, with three of them involved in G-protein coupled receptor (GPCR) signaling (Figure 4).

One of the hypermethylated pathways found to be enriched in LTS from the TMZ group with m-MGMT was WNT signaling. We wanted to check whether there was a correlation between methylation of DMGs from the enrichment core and the expression of these, but gene expression data were not available for our samples. Instead, we used 51 primary, *IDH* wildtype GBM samples with RNA-seq and 450k methylation array data from TCGA, accessed via the SMART App website (Li et al., 2019). First, we identified which of the DMCs from the enrichment core were also covered by the 450k beadchip arrays, since our results were based on the newer design, the 850k methylation array. The overlapping DMCs were found for nine genes, which were then analyzed with the Spearman correlation coefficient to find if the promoter methylation status of the genes correlated with the corresponding mRNA expression, at a significance level *p* < 0.05. A negative correlation was found only for the *WNT2* gene (R = -0.37, *p* = 0.0067).

### Epigenetic age

**FIGURE 5:**
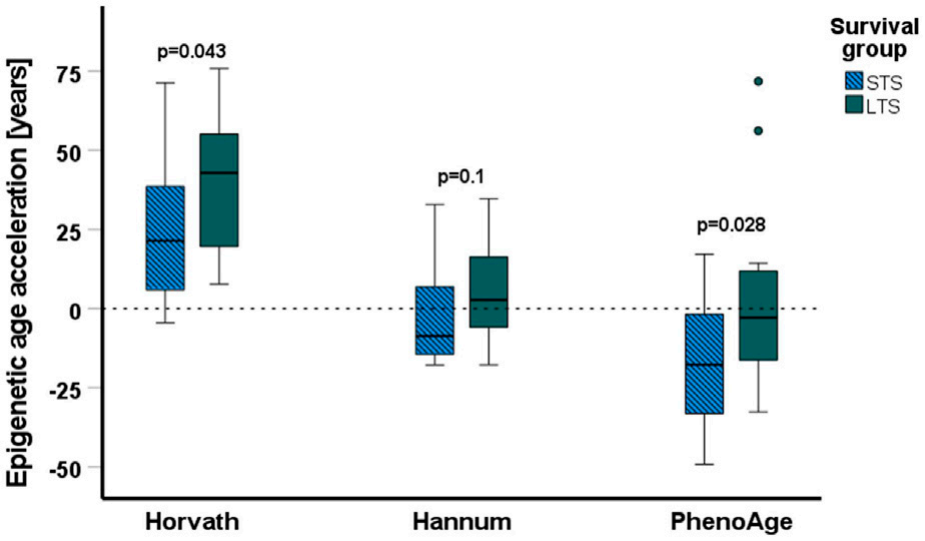
Results of the epigenetic age acceleration analyses. The epigenetic age acceleration (difference between the epigenetic age and the chronological age) is shown in years on the *y*-axis, horizontal lines represent the median values, boxplots represent the first and third quartiles, errors are represented by the T-bars, and the dots represent outliers. Epigenetic age acceleration is significantly lower for short-term survivors (STS) (*N* = 18) than long-term survivors (LTS) (*N* = 18) when calculated with Horvath’s and PhenoAge algorithms. All data above the dashed line represent an acceleration of epigenetic age, and data below the dotted line represent a deceleration of epigenetic age.

The cofactor analysis showed that chronological age did not affect DMCs (Supplementary Data, Supplementary Figure S2); however, molecular alterations in cancer cells may affect the epigenetic age. This prompted us to analyze the epigenetic age of the groups consisting of three tumor samples, which involved three different epigenetic clocks, Horvath’s (Horvath, 2013), Hannum’s (Hannum et al., 2013), and PhenoAge (Levine et al., 2018). The calculated epigenetic age was compared to the chronological age of the patient to establish epigenetic age acceleration. We found significant differences between mean epigenetic age acceleration calculated with Horvath’s (STS vs. LTS; 23.6 vs. 39.2 years, *p* = 0.043) and PhenoAge (STS vs. LTS; -18 vs. 0.2 years, *p* = 0.028) epigenetic clocks of STS and LTS in the analysis that combined samples from all treatment arms and with both methylated and unmethylated *MGMT* promoter (Figure 5). As the number of samples analyzed was small, i.e., only 18 in all, to further statistically improve our finding of the prognostic value of epigenetic age acceleration for the Horvath’s clock in an analysis regarding LTS and STS for patients with the same characteristics as used in this study, it would be necessary to include at least 32 samples per group to maintain a power of 80% with alpha equal to 0.05. However, it is important to keep in mind that samples used here were selected based on their significant survival difference, and these power calculations do not include the midpoint survival. To analyze whether our findings were treatment- or *MGMT* promoter methylation status-specific, we also compared the epigenetic age and epigenetic age acceleration between STS and LTS in the groups defined by these factors. According to Horvath’s algorithm, all samples had a higher epigenetic than chronological age (Supplementary Data, Supplementary Table S3), but there was no significant difference between the epigenetic age in LTS and STS, when separated into different treatment arms with different *MGMT* promoter methylation statuses. There were also no differences in the age acceleration (Supplementary Data, Supplementary Table S3). In the results from Hannum’s and PhenoAge epigenetic clocks, both epigenetic age acceleration and deceleration (epigenetic age < chronological age) were observed, with STS groups usually characterized by lower mean epigenetic age (Supplementary Data, Supplementary Table S3). Significant differences were discovered only in the 34Gy, u-MGMT group (Hannum’s and PhenoAge) and in the combined RT, u-MGMT group (Hannum’s). In these cases, STS showed epigenetic age deceleration and lower mean epigenetic ages than LTS.

## Discussion

In recent years an increase in the use of methylome profiling has been observed. The widely known brain tumor classifier proposed by Capper et al. (2018) allows for a more precise classification of brain malignancies. Undoubtedly, methylation of the *MGMT* promoter remains the most important biomarker for GBM, which predicts the effect of TMZ treatment (Stupp et al., 2007; Malmstrom et al., 2012; Weller et al., 2020). However, we lack validated predictive biomarkers for patients with u-MGMT tumors or for those treated with RT. Here, we employed the Capper classifier (Capper et al., 2018) and differential methylation to identify biomarkers predisposing to good treatment response or indicating resistance to therapy.

Inter- and intratumor heterogeneity of GBM plays an important role in treatment resistance and relapse of the disease. A previous study on spatially separated biopsies showed that methylation heterogeneity is common in GBM, though it seems to affect only the subclassification, whilst all samples remain classified as GBM *IDH* wildtype and *MGMT* methylation status also remains stable (Wenger et al., 2019). This is in line with our results from the methylation-based classification, which showed that in 34 samples more than one subclass was identified. Interestingly, it has recently been reported that treatments such as chemo- or radiotherapy, and even hypoxia, as sources of stress for cancer cells, may induce methylation changes that likely contribute to the heterogeneity among tumor cells (Johnson et al., 2021) and possibly patients’ outcome.

In LTS treated with TMZ and with m-MGMT, our analysis revealed that the WNT signaling pathway was hypermethylated, implicating silencing of WNT signaling and indicating the importance of a maintained WNT pathway activity in STS. This aligns with the previously presented results by Shinawi et al. (2013), in which hypermethylation of the promoter of the *DKK2* gene, an antagonist of the WNT pathway, was found in STS. In TCGA data, we found a negative correlation between methylation of the sites corresponding to hypermethylated CpGs in our samples and the expression of the *WNT2* gene. In mice, WNT2 drives the proliferation of progenitor cells and development of the midbrain, possibly having similar effects in humans (Sousa et al., 2010). Wnt signaling is crucial during embryonal development and is commonly altered in cancers (Zhan et al., 2017). In GBM, activation of the WNT pathway is necessary for the maintenance of glioblastoma cancer stem cells, which greatly contribute to treatment resistance (Lee et al., 2016). Importantly, methylation may be the main regulatory mechanism of the WNT pathway in GBM, as mutations are rare (Lee et al., 2016). GBM is also proposed to originate from the subventricular zone (SVZ), an area supporting neural progenitor cells (Lee et al., 2018), and tumor proximity to the SVZ has been linked to STS (Adeberg et al., 2014). Methylation of gene promoters in newly diagnosed GBM and their impact on gene expression, and survival of patients treated with concomitant radiochemotherapy, was also previously reported by Etcheverry et al. (2010). They found six CpG sites, for which methylation was associated with decreased survival, and two of the CpG sites were localized in the *SOX10* gene promoter. We did not find *SOX10* among DMGs in any of the treatment arms, but active WNT signaling acts inhibitory on *SOX10* expression (Uka et al., 2020). To further emphasize this signaling pathway, a study by Wu et al. (2020) showed that SOX10 acts as a master regulator of the receptor tyrosine kinase I (RTK1) subtype of GBM, which often harbors platelet-derived growth factor receptor alpha amplification (Wu et al., 2020).

Another interesting finding, also in LTS with m-MGMT promoter treated with TMZ is hypermethylation of the platelet activation, signaling, and aggregation pathway. Up to 30% of GBM patients experience venous thromboembolism, making it one of the most common and serious complications, which may influence survival (Yust-Katz et al., 2015; Heenkenda et al., 2019). Hypermethylation of the platelet activation pathway may therefore have a protective function.

Among the hypomethylated enriched pathways, we found some that were labeled as neuronal system, neurotransmitter receptors, transmission across chemical synapses, and potassium channels, all of which have been shown to partake in GBM development and progression (Venkataramani et al., 2019; Venkatesh et al., 2019). In our study, however, these pathways are associated with LTS treated with TMZ and with m-MGMT. It should be noted that in most of the published studies mentioned here, STS is referred to patients with a survival of <1 year and LTS to those with a survival of >3 years. In our study of elderly patients with GBM, median survival was 12 months, and the survival range was 4.5–126 months for the most favorable group, those treated with TMZ and m-MGMT.

TMZ is the most commonly used drug in GBM treatment due to its good blood–brain barrier penetration and generally mild toxicity (Stupp et al., 2005), and *MGMT* promoter methylation status is predictive for the treatment response to TMZ (Malmstrom et al., 2012). However, not all GBMs are tested for *MGMT* (Malmstrom et al., 2020). Chai et al. (2019) investigated the potential benefits of TMZ in patients with u-MGMT and showed that by using a methylation signature of 31 genes, it is possible to select patients with u-MGMT to obtain a survival similar to that of patients with m-MGMT. Unfortunately, we did not find any DMCs separating LTS and STS in the TMZ arm with u-MGMT.

In LTS from the 34Gy treatment arm with m-MGMT promoter, the enriched pathway in the hypermethylated DMGs is the pathway of the secretin family receptors, a subgroup of G-protein coupled receptors (GPCRs). Pathways involving GPCR are enriched in the TMZ and 34Gy arms when filtering conditions are less stringent. GPCR is the largest family of membrane proteins involved in cell metabolism, migration, neurotransmission, immune response, and cell differentiation (Byrne et al., 2021). Hypermethylation of these pathways may lead to downregulation of gene expression and decreased activity, potentially having a protective effect on the patients. In fact, GPCRs are explored in various studies as treatment targets for glioblastoma (Byrne et al., 2021), but in our limited study, we did not find any DMCs in the combined RT group to indicate the importance of methylation of GPCR for RT outcome.

The magnitude and direction of epigenetic age changes were dependent on the applied algorithm, but overall, we observed a trend toward lower epigenetic age in STS in comparison to LTS in all algorithms. We found that STS had a significantly lower acceleration of epigenetic age than LTS in the combination groups from all treatment arms and when calculated with Horvath’s and PhenoAge algorithms. Only Hannum’s clock failed to show a significant difference. The discrepancies between the results are likely caused by the innate differences between the algorithms. Horvath’s clock pioneered the field and can be universally used for different tissues (Horvath, 2013), Hannum’s clock is primarily designed for the assessment of epigenetic age from blood, and the PhenoAge model was built on phenotypic age, including many morbidity- and mortality-related factors. Hannum’s method and PhenoAge both showed deceleration of epigenetic age instead of acceleration, which is possibly dictated by the tissues and factors used for model development. However, the results from Horvath’s clock are in line with previous studies performed on gliomas, indicating acceleration of epigenetic age in tumor tissue (Liao et al., 2018; Zheng et al., 2020). The surprising effect of epigenetic age deceleration or lower age in STS could be the result of the involvement of stem-like cells in GBM development and progression, since stem cells are characterized by lower epigenetic age (Horvath, 2013). Although we did not find consistent and significant differences in the epigenetic age of LTS and STS in most of the analyzed treatment groups, this was probably caused by the low number of samples. Overall, we observed a general distortion in the epigenetic age in comparison to the chronological age, which could be further explored as a prognostic factor. A very recent study, which included four datasets, one of them being the Nordic trial analyzed with the Illumina 450k array, showed a 70% overlap between CpGs used for methylation-based classification and sites linked to epigenetic age acceleration (Bady et al., 2022). The RTKII subclass exhibited the highest epigenetic age acceleration in comparison to the RTKI and MES tumors. Due to the limited number of samples included here, we were not able to conduct the same comparison with our 850k-generated data. At the same time, the mentioned study reported no differences in the results related to younger and older patients, indicating a lack of relevance of chronological age in relation to the epigenetic age of the GBM tissue (Bady et al., 2022).

The value of methylome profiling for brain tumors has been largely shown through the methylation-based CNS classifier (Capper et al., 2018). Although our analyses are limited due to the number of samples, they highlight methylation differences, also related to treatment modality, that exist between LTS and STS with GBM, which might be clinically relevant. Also, epigenetic age assessment may potentially be a valuable tool to select patients with good prognosis. However, systematic analysis of larger cohorts of patients with LTS and STS is necessary and warranted for validation of our findings.

## Data Availability

The datasets generated for this study can be found in the Gene Expression Omnibus database (GEO) (http://www.ncbi.nlm.nih.gov/geo/) under the accession number GSE207426.
