# Supplementary material for: Methylation associated with long- or short-term survival in glioblastoma patients from the Nordic phase 3 trial
Source: Front Genet. 2022 Aug 25;13:934519. doi: 10.3389/fgene.2022.934519 (PMC9452748; doi:10.3389/fgene.2022.934519)
Supplement: Supplementary file 1 [file DataSheet1.docx]

Supplementary Material

# Supplementary Figures and Tables

## Supplementary Figures


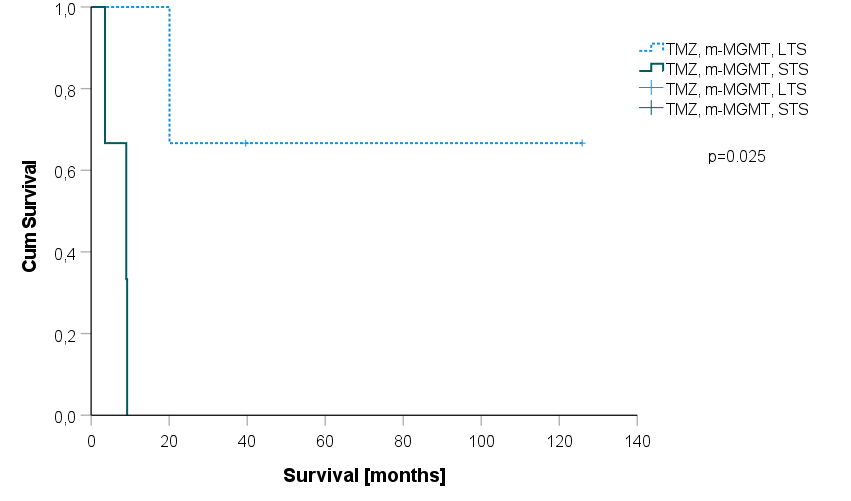
A


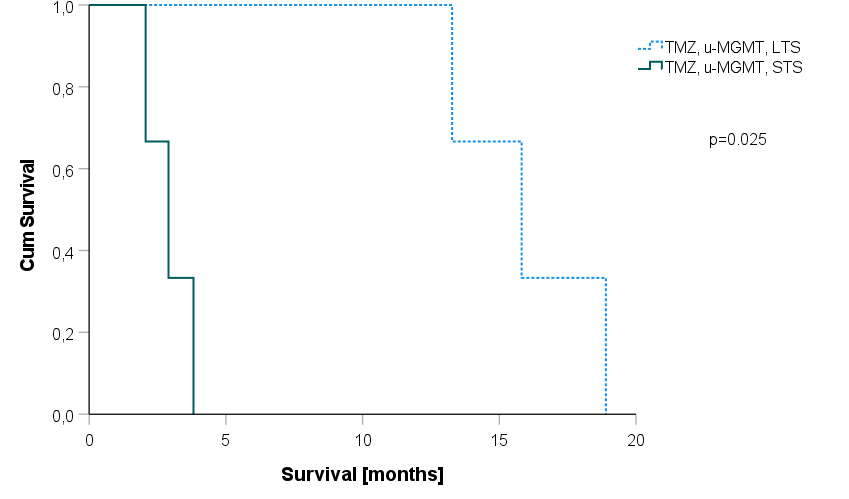
B


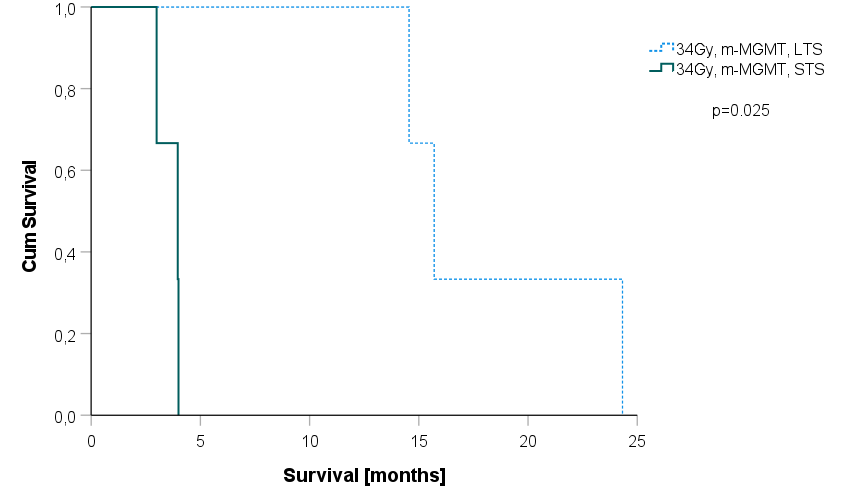
C

D


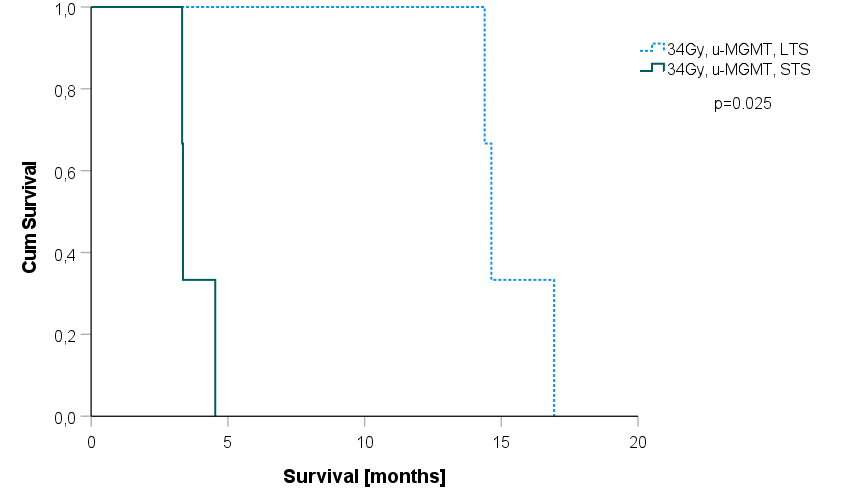


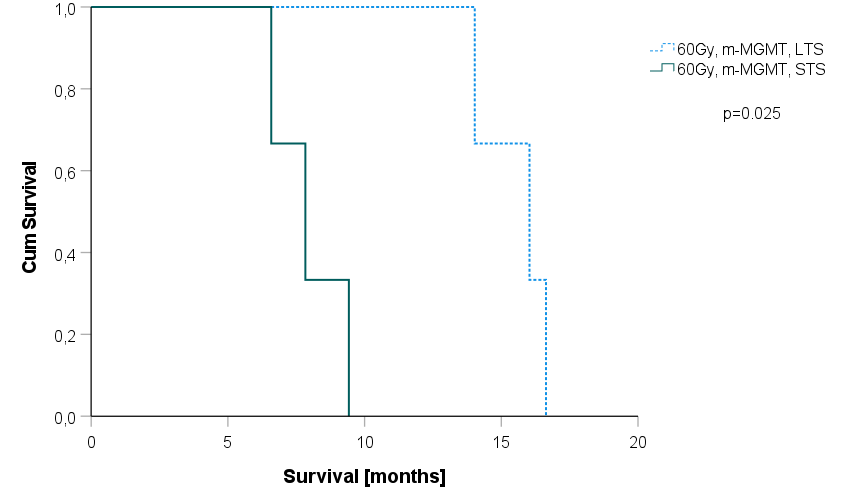
E


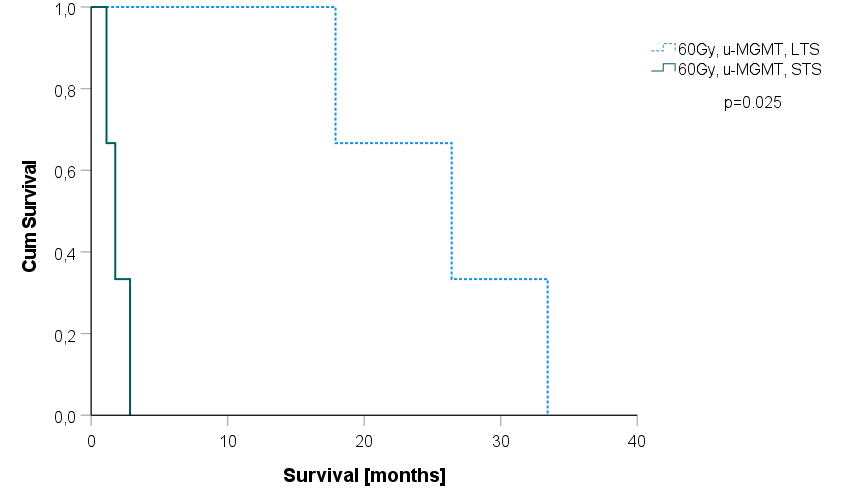
F

**
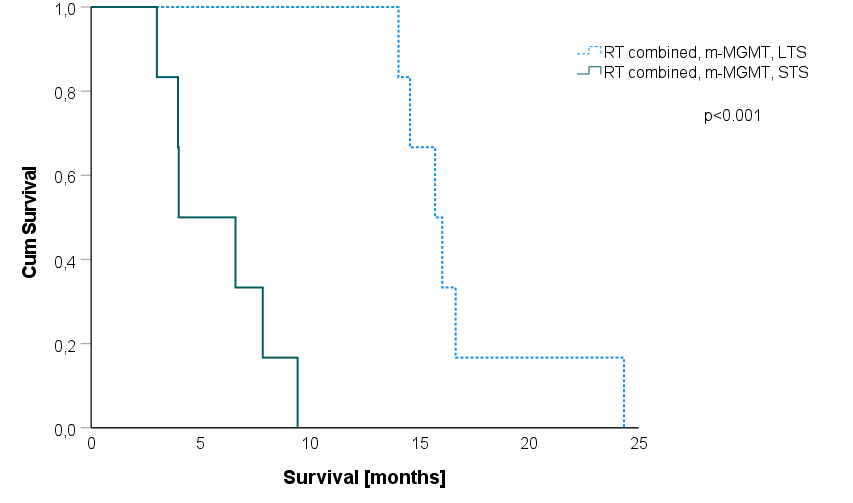
**G


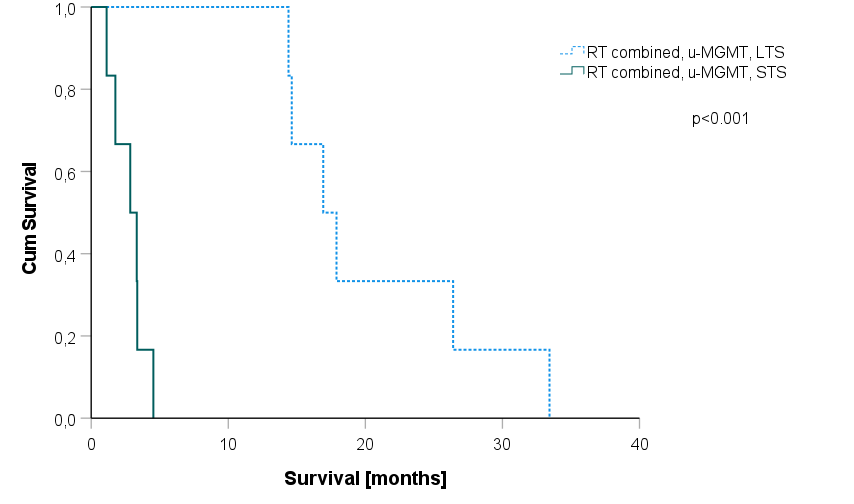
H

**Supplementary Figure S1.** Kaplan-Meier curves with log rank statistics comparing LTS and STS of all treatment modalities in combination with MGMT status. The analysis included samples used for DMCs discovery and epigenetic age calculations. Statistical significance was considered at 95%.


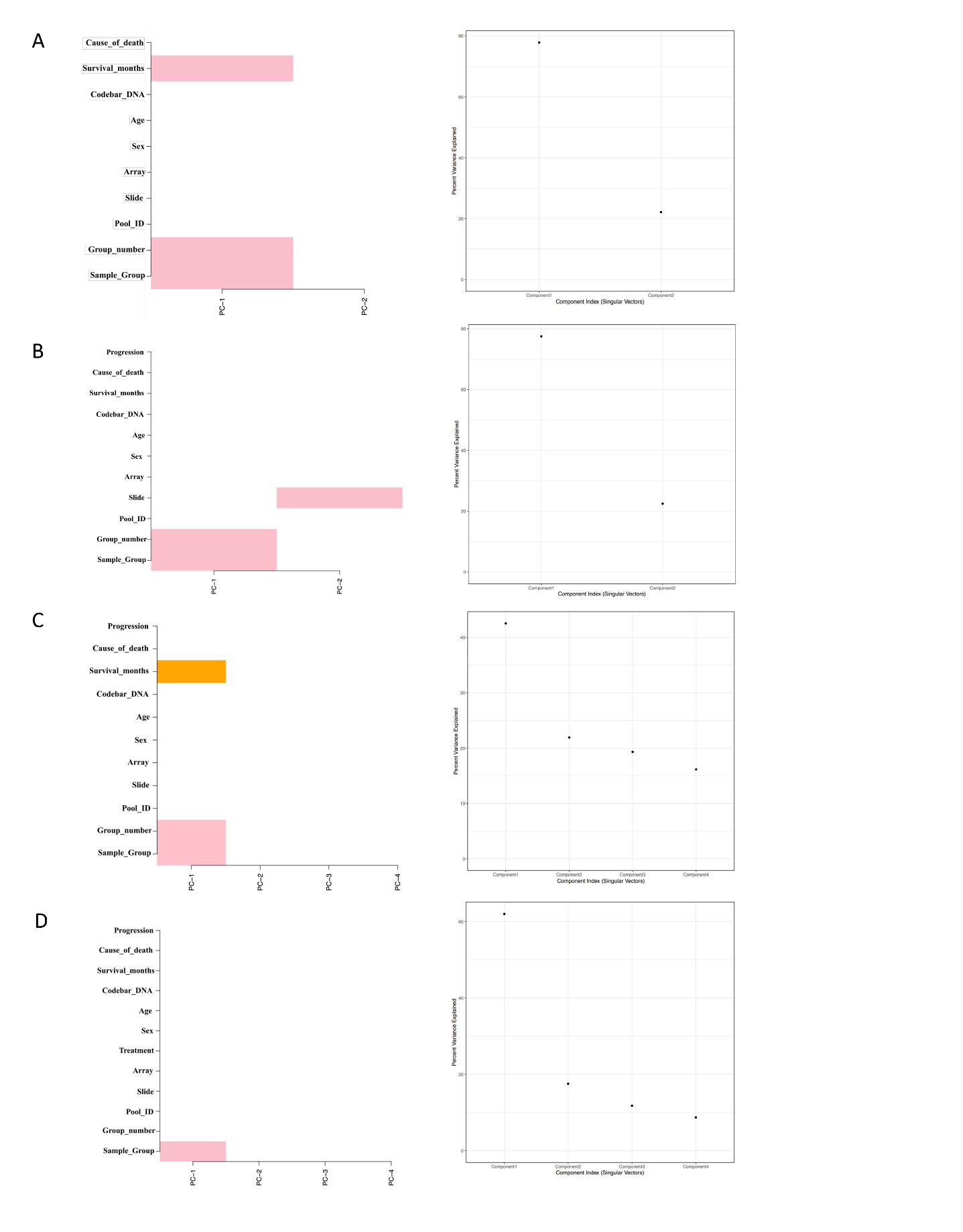


**Supplementary Figure S2.** Results of the SVD analysis for groups with discovered DMCs; TMZ, m-MGMT (A), 34Gy, m-MGMT (B), 60Gy, u-MGMT (C), combined RT, u-MGMT (D).
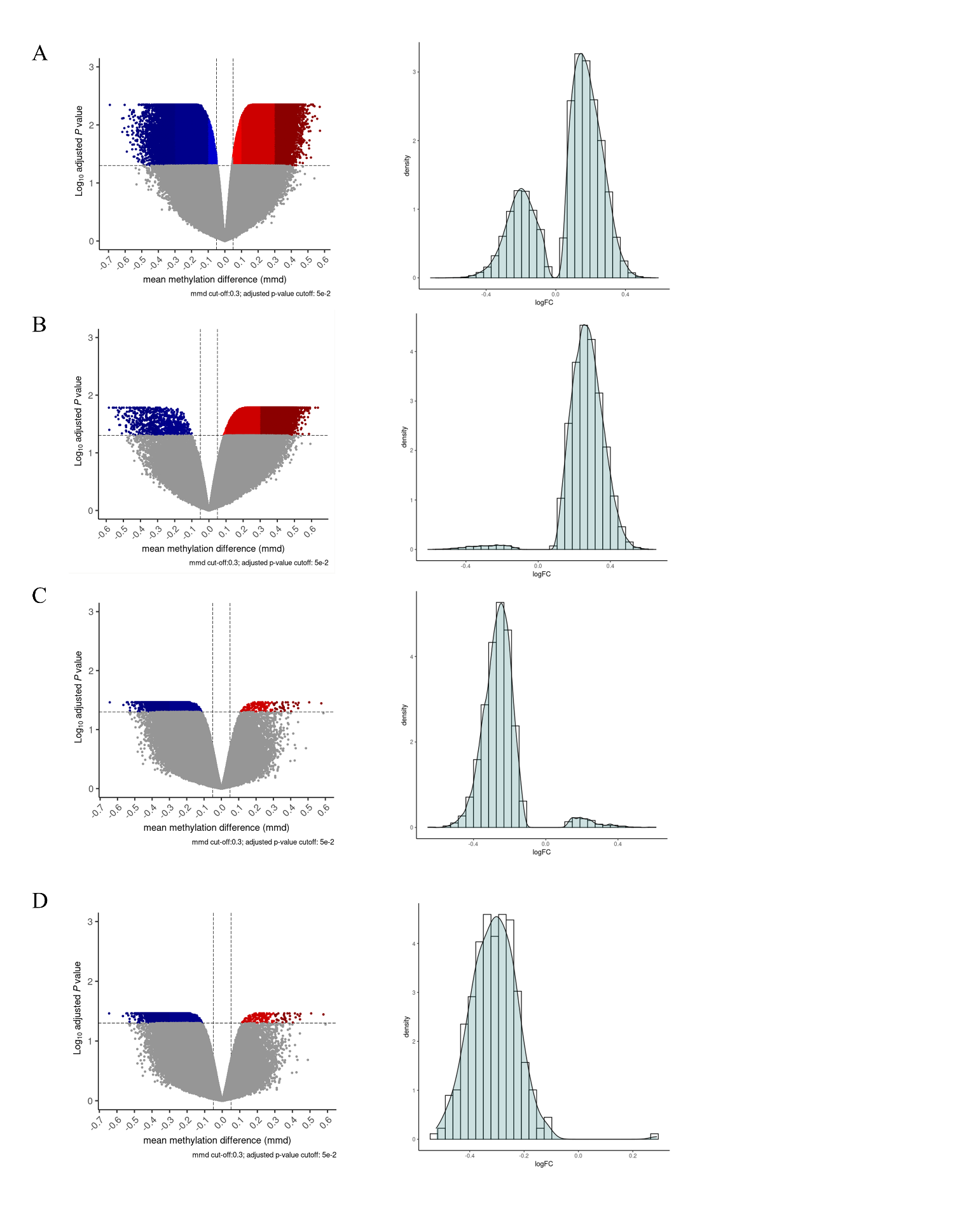


**Supplementary Figure S3.** Volcano plots (left side) and density plots (right side) representing DMCs from TMZ, m-MGMT (A), 34Gy, m-MGMT (B), 60Gy, u-MGMT (C), RT, u-MGMT (D). Colored dots in the volcano plots in the darkest shades of blue and red represent DMCs fulfilling the cut-off requirements (*p*-value_BH_ < 0.05; (∆mmd) ≥|0.3|). Hypermethylated DMCs have positive values on the logFC axis in the density plots and hypomethylated DMCs have negative values logFC.

## Supplementary Tables

**Supplementary Table S1.** Number of differentially methylated CpGs (DMCs) and genes (DMGs) before and after location-based filtration.

| **Comparison group (LTS vs. STS)** | **Differentially methylated CpGs** | | | | |
| --- | --- | --- | --- | --- | --- |
|  | **before filtering** | | **only located at TSS and 5’UTR** | | |
|  | **DMCs** | **DMGs** | **DMCs** | **DMGs** |  |
| **TMZ, m-MGMT** | 123510 | 18344 | 26626 | 11443 |  |
| **34Gy, m-MGMT** | 39649 | 1104 | 39649 | 4915 |  |
| **60Gy, u-MGMT** | 4086 | 1961 | 4086 | 816 |  |
| **Combined RT, u-MGMT** | 319 | 181 | 319 | 82 |  |

**Supplementary Table S2.** Results of Reactome pathway enrichment analysis for filtered DMGs.

| **Reactome ID** | **Reactome Pathway** | **Enrichment score** | **Adjusted *p*-value** |
| --- | --- | --- | --- |
| **TMZ, m-MGMT, hypermethylated DMGs** | | | |
| R-HSA-1430728 | Metabolism | 0.2310 | 4x10^-2^ |
| R-HSA-76002 | Platelet activation, signaling and aggregation | 0.4427 | 4x10^-2^ |
| R-HSA-162582 | Signal Transduction | 0.1964 | 4x10^-2^ |
| R-HSA-195721 | Signaling by WNT | 0.5736 | 4x10^-2^ |
| **TMZ, m-MGMT, hypomethylated DMGs** | | | |
| R-HSA-168256 | Immune System | 0.2813 | 2x10^-7^ |
| R-HSA-162582 | Signal Transduction | 0.2006 | 9x10^-6^ |
| R-HSA-168249 | Innate Immune System | 0.2994 | 2x10^-4^ |
| R-HSA-5653656 | Vesicle-mediated transport | 0.3626 | 2x10^-3^ |
| R-HSA-597592 | Post-translational protein modification | 0.2861 | 2x10^-3^ |
| R-HSA-1643685 | Disease | 0.2282 | 2x10^-3^ |
| R-HSA-2682334 | EPH-Ephrin signaling | 0.6375 | 2x10^-3^ |
| R-HSA-199991 | Membrane Trafficking | 0.3641 | 2x10^-3^ |
| R-HSA-1280215 | Cytokine Signaling in Immune system | 0.2969 | 2x10^-3^ |
| R-HSA-5663205 | Infectious disease | 0.2796 | 2x10^-3^ |
| R-HSA-109582 | Hemostasis | 0.2902 | 2x10^-3^ |
| R-HSA-392499 | Metabolism of proteins | 0.2262 | 3x10^-3^ |
| R-HSA-6798695 | Neutrophil degranulation | 0.3438 | 3x10^-3^ |
| R-HSA-112314 | Neurotransmitter receptors and postsynaptic signal transmission | 0.3745 | 3x10^-3^ |
| R-HSA-112315 | Transmission across Chemical Synapses | 0.3340 | 3x10^-3^ |
| R-HSA-1280218 | Adaptive Immune System | 0.2992 | 6x10^-3^ |
| R-HSA-112316 | Neuronal System | 0.2673 | 6x10^-3^ |
| R-HSA-74160 | Gene expression (Transcription) | 0.2246 | 6x10^-3^ |
| R-HSA-9675108 | Nervous system development | 0.2727 | 9x10^-3^ |
| R-HSA-212436 | Generic Transcription Pathway | 0.2258 | 9x10^-3^ |
| R-HSA-73857 | RNA Polymerase II Transcription | 0.2258 | 9x10^-3^ |
| R-HSA-1266738 | Developmental Biology | 0.2040 | 1x10^-2^ |
| R-HSA-422475 | Axon guidance | 0.2704 | 1x10^-2^ |
| R-HSA-202733 | Cell surface interactions at the vascular wall | 0.4847 | 2x10^-2^ |
| R-HSA-9006934 | Signaling by Receptor Tyrosine Kinases | 0.2480 | 2x10^-2^ |
| R-HSA-1296071 | Potassium Channels | 0.4801 | 2x10^-2^ |
| R-HSA-977443 | GABA receptor activation | 0.4791 | 2x10^-2^ |
| R-HSA-194315 | Signaling by Rho GTPases | 0.2796 | 2x10^-2^ |
| R-HSA-9716542 | Signaling by Rho GTPases, Miro GTPases and RHOBTB3 | 0.2796 | 2x10^-2^ |
| R-HSA-397014 | Muscle contraction | 0.3594 | 2x10^-2^ |
| R-HSA-556833 | Metabolism of lipids | 0.3 | 2x10^-2^ |
| R-HSA-449147 | Signaling by Interleukins | 0.3006 | 3x10^-2^ |
| R-HSA-2029480 | Fcgamma receptor (FCGR) dependent phagocytosis | 0.4053 | 3x10^-2^ |
| R-HSA-1430728 | Metabolism | 0.1621 | 3x10^-2^ |
| R-HSA-1500931 | Cell-Cell communication | 0.4229 | 4x10^-2^ |
| R-HSA-9658195 | Leishmania infection | 0.2667 | 4x10^-2^ |
| R-HSA-418346 | Platelet homeostasis | 0.4401 | 4x10^-2^ |
| **34Gy, m-MGMT, hypermethylated DMGs** | | | |
| R-HSA-168256 | Immune System | 0.1954 | 4x10^-2^ |
| R-HSA-373080 | Class B/2 (Secretin family receptors) | 0.6234 | 4x10^-2^ |

**Supplementary Table S3.** Results of the epigenetic age calculations and comparisons of mean ages between LTS and STS.

| Treatment | MGMT status | Biological age [LTS vs. STS; p] | Horvath [LTS vs. STS; p] | Hannum [LTS vs. STS; p] | PhenoAge [LTS vs. STS; p] | Horvath acceleration [LTS vs. STS; p] | Hannum acceleration [LTS vs. STS; p] | PhenoAge acceleration [LTS vs. STS; p] |
| --- | --- | --- | --- | --- | --- | --- | --- | --- |
| TMZ | Methylated | 65 vs. 69; p=0.23 | 98.5 vs. 82.9; p=0.454 | 65.9 vs. 62.3; p=0.725 | 60.5 vs. 51.8; p=0.69 | 33.5 vs. 13.9; p=0.33 | 0.9 vs. -6.7; p=0.443 | -4.5 vs.  -17.2; p=0.54 |
|  | Unmethylated | 73.3 vs. 67.7; p=0.086 | 97.3 vs. 81.4; p=0.48 | 68.9 vs. 67.7; p=0.936 | 62.6 vs. 39.6; p=0.187 | 24 vs. 13.7; p=0.629 | -4.5 vs. 0.1; p=0.712 | -10.7 vs. -28.1; p=0.258 |
|  | Methylated+ unmethylated | 69.2 vs. 68.3; p=0.749 | 97.9 vs. 82.1; p=0.234 | 67.4 vs. 65; p=0.756 | 61.6 vs. 45.7; p=0.196 | 28.8 vs. 13.8; p=0.242 | -1.8 vs.  -3.3; p=0.827 | -7.6 vs.  -22.6; p=0.19 |
| 34Gy | Methylated | 69.3 vs. 69.3; p=1.0 | 107.2 vs. 99; p=0.584 | 66.2 vs. 77.2; p=0.556 | 79.3 vs. 64.1; p=0.639 | 37.8 vs. 29.6; p=0.537 | -3.1 vs. 7.9; p=0.505 | 9.9 vs. -5.3; p=0.593 |
|  | Unmethylated | 70.3 vs. 67.7; p=0.294 | 115.4 vs. 83.3; p=0.078 | 76.7 vs. 53.8; p=0.021* | 68.9 vs. 41.1; p=0.02* | 45 vs. 15.6; p=0.073 | 6.4 vs. -13.9; p=0.023* | -1.4 vs. -26.6; p=0.036* |
|  | Methylated+ unmethylated | 69.8 vs. 68.5; p=0.581 | 111.3 vs. 91.1; p=0.061 | 71.4 vs. 65.6; p=0.568 | 74.1 vs. 53.6; p=0.18 | 41.4 vs. 22.6; p=0.051 | 1.6 vs. -3; p=0.621 | 4.3 vs.  -15.9; p=0.163 |
| 60Gy | Methylated | 60.3 vs. 71.7; p=0.0003* | 107.7 vs. 106; p=0.958 | 83.7 vs. 73.3; p=0.442 | 82.8 vs. 55; p=0.389 | 47.3 vs. 34.4; p=0.671 | 23.4 vs. 1.6; p=0.156 | 22.5 vs. -16.7; p=0.251 |
|  | Unmethylated | 66.7 vs. 68; p=0.735 | 114.2 vs. 102.5; p=0.649 | 78.2 vs. 63.1; p=0.361 | 52 vs. 54; p=0.931 | 47.6 vs. 34.5; p=0.603 | 11.5 vs. -4.9; p=0.318 | -14.7 vs.  -14.1; p=0.977 |
|  | Methylated+ unmethylated | 63.5 vs. 69.8; p=0.023* | 111 vs. 104.3; p=0.696 | 80.9 vs. 68.2; p=0.183 | 67.4 vs. 54.5; p=0.474 | 47.5 vs. 34.5; p=0.444 | 17.4 vs.  -1.6: p=0.061 | 3.9 vs.  -15.4; p=0.307 |
| Combined RT (34Gy+60Gy) | Methylated | 64.8 vs. 70.5; p=0.085 | 107.4 vs. 102.5; p=0.735 | 74.9 vs. 75.3; p=0.977 | 81 vs. 59.5; p=0.277 | 42.6 vs. 32; p=0.466 | 10.1 vs. 4.7; p=0.627 | 16.2 vs.  -11; p=0.161 |
|  | Unmethylated | 68.5 vs. 67.8; p=0.756 | 114.8 vs. 93; p=0.124 | 77.4 vs. 58.5; p=0.028* | 60.5 vs. 47.5; p=0.27 | 46.3 vs. 25; p=0.119 | 8.9 vs.  -9.36; p=0.031* | -8 vs. -20.3; p=0.269 |
|  | Methylated+ unmethylated | 66.7 vs. 69.1; p=0.192 | 111.1 vs. 97.7; p=0.165 | 76.1 vs. 66.9; p=0.169 | 70.8 vs. 53.5; p=0.131 | 44.4 vs. 28.5; p=0.091 | 9.5 vs. -2.3; p=0.084 | 4.1 vs.  -15.7; p=0.078 |

*p<0.05
